# Cancer Prediction Nomograms for Advanced Practitioners in Oncology

**Published:** 2014-09-01

**Authors:** Lydia T. Madsen

**Affiliations:** Dr. Madsen is an advanced practice nurse at MD Anderson Cancer Center in Houston, Texas.

Cancer prediction nomograms provide advanced practitioners (APs) with tools to assess a patient’s overall risk for development of a specific cancer. Alternatively, after a cancer diagnosis has been made, nomograms represent a standardized means to provide a statistical estimate of prognosis, particularly in individuals with characteristics of high-risk disease. According to the National Cancer Institute (NCI), these prediction models are a means to "facilitate the design and planning of clinical cancer trials, foster the development of benefit-risk indices, and enable estimates of the population burden and cost of cancer" (NCI, 2014a).

## Patient Education and Appropriate Use of Nomograms

As APs in oncology, we frequently educate patients on the risks of current or recurrent disease status. Incorporating these cancer prediction nomograms, or "prediction tools," into patient education may also provide supportive statistical evidence for patients to make knowledgeable decisions regarding treatment. It is important to note that cancer prediction nomograms are supportive tools for use in both treatment planning and as a part of a comprehensive education on best evidence for patients.

Predictive nomograms are based on the statistical probability of disease risk on the basis of identified characteristics and are not intended for use as a substitute for physician diagnosis. Patients should be cautioned that nomograms should not be used as the sole means to determine risk for development of disease or to recommend treatment for a specific type of cancer but rather for use as a tool in the comprehensive care management of any patient (Memorial Sloan Kettering Cancer Center [MSKCC], 2014).

The list below is a compilation of selected risk calculators associated with several common cancers. They are based on statistical modeling from large patient databases and represent current best evidence for disease-specific risk of cancer development and recurrence or for prognosis.

## Breast and Ovarian Cancer

**Breast Cancer Risk Assessment Tool (NCI)**

http://www.cancer.gov/bcrisktool/

Women can use the NCI’s Breast Cancer Risk Assessment Tool—which is based on a statistical model known as the Gail model—independently, or APs can use it as a teaching tool. Based on personal medical history, family history of breast cancer (immediate, first-degree relatives), and an individual’s reproductive history, the risk for developing invasive breast cancer is calculated and based on statistical modeling. This tool has been extensively tested. It provides an accurate estimate of breast cancer risk, although it may underestimate the risk of developing breast cancer for African American women.

**The BOADICEA Model of Genetic Susceptibility to Breast and Ovarian Cancers (University of Cambridge)**

http://ccge.medschl.cam.ac.uk/boadicea/boadicea-web-application

The BOADICEA (Breast and Ovarian Analysis of Disease Incidence and Carrier Estimation Algorithm) model is a risk assessment tool developed to estimate the probability of developing breast or ovarian cancer associated with mutations in BRCA1 and BRCA2. This tool is not easily accessed; it requires enrollment in the site. But because this prediction nomogram is intended to estimate the possibility of cancer susceptibility gene mutation specific to families or individuals, it may be particularly useful for screening those considering genetic testing (NCI, 2014b).

## Prostate Cancer

**Prostate Cancer Nomogram: Pretreatment (MSKCC)**

http://nomograms.mskcc.org/Prostate/PreTreatment.aspx

The MSKCC risk assessment for prostate cancer recurrence (pretreatment) is the most commonly used nomogram for patients initially diagnosed with prostate cancer. This nomogram helps patients to understand the statistical probability, in percentages, of having disease that will recur based on the treatment they elect to undergo at the time of diagnosis. It is based on the clinical information obtained at the time of diagnosis, such as Gleason score, prostate-specific antigen (PSA) value, number of positive and negative biopsy cores, and stage of disease on examination.

Although patients can input this information themselves, it is most valuable when discussed with a clinician who can assist with the interpretation of the results. In contrast to the Gail model, this particular nomogram provides more detail than a statistical percentage: It provides percentage risk for a variety of factors, including involvement of extracapsular areas, seminal vesicles, and lymph nodes. Advanced practitioners can use these percentages to compare treatment options with patients. Interpreting the nomogram results and discussing the implications in great detail may be beneficial in assisting patients with their treatment decisions.

**Prostate Cancer Nomogram: Male Life Expectancy (MSKCC)**

http://nomograms.mskcc.org/Prostate/LifeExpectancy.aspx

The MSKCC prostate cancer calculation of male life expectancy is intended for use as a tool when considering various treatment options. Although patients often receive information regarding the advantages and limitations of surgery vs. radiation therapy, or active surveillance, calculating life expectancy may add additional information on which to base important treatment decisions.

## GIST

**Gastrointestinal Stromal Tumor Nomogram (MSKCC)**

http://nomograms.mskcc.org/GastroIntestinal/ GastroIntestinalStromalTumor.aspx

The MSKCC gastrointestinal stromal tumor (GIST) nomogram is intended for use in patients with a known diagnosis of GIST. The tool calculates the statistical chance of survival without adjuvant or neoadjuvant therapy and is intended to provide a statistical estimate of the success of surgery alone as treatment.

## Melanoma

**Individualized Melanoma Patient Outcome Prediction Tools:**

**Localized or Regional Disease (AJCC)**

www.melanomaprognosis.org

The Individualized Melanoma Patient Outcome Prediction Tools are intended to assist in predicting the clinical prognosis of patients at the time of initial diagnosis (American Joint Committee on Cancer [AJCC], 2010). There is one tool for patients with localized disease and another for those with regionally metastatic disease at the time of diagnosis. Based on individual clinical and pathologic information, the nomogram can predict survival rates at 1, 5, and 10 years at a 95% confidence level.

## Kidney Cancer

**Kidney Cancer Predictive Tools (Fox Chase Cancer Center)**

http://labs.fccc.edu/nomograms/main.php?nav=3&audience=1

The kidney cancer prediction nomograms from Fox Chase are comprehensive and pathway specific (Fox Chase Cancer Center, 2014). The kidney function nomogram was developed to predict the statistical likelihood of developing chronic kidney disease based on age and comorbidities. The website for predictive nomograms includes the following:

1. A prognostic preoperative model to calculate statistical risk of recurrence of renal cell carcinoma (RCC) after surgery; an assessment for risk of high-grade malignancy based on preoperative variables is also included;
2. A prognostic postoperative model designed to predict 1-, 2-, and 5-year recurrence-free, disease-specific, and overall survival based on variables;
3. Prognostic calculators for statistical likelihood of (a) survival in patients with metastatic RCC based on risk stratification at diagnosis if receiving traditional immunotherapy or (b) 12-month progression-free survival in patients with clear cell RCC pathology receiving sunitinib (Sutent).

## Testis Cancer

**Testis Cancer Predictive Tools (Fox Chase Cancer Center)**

http://labs.fccc.edu/nomograms/main.php?nav=5&audience=1

These two tools from Fox Chase provide a statistical estimate of the following:

1. Likelihood of a complete response to platinum-based chemotherapy, based on the histology, site of disease, extent of disease, and baseline markers at diagnosis for patients with testicular cancer;
2. Prognosis for patients who progress after platinum-based chemotherapy; specific to individuals with a nonseminomatous germ cell pathology and is calculated based on time to relapse, whether the response to chemotherapy was complete or incomplete, and markers present at the time of relapse.

## Further Resources

For detailed information on the current nomograms available for a variety of diseases, the Epidemiology and Genomics Research Program at the NCI maintains a comprehensive listing of those in current use or under development (NCI, 2014c). This site is an up-to-date resource for APs; it contains a comprehensive listing of peer-reviewed risk-prediction models that can be beneficial teaching tools for current oncology practice. l
